# Using parametric regressors to disentangle properties of multi-feature processes

**DOI:** 10.1186/1744-9081-4-38

**Published:** 2008-08-15

**Authors:** Guilherme Wood, Hans-Christoph Nuerk, Denise Sturm, Klaus Willmes

**Affiliations:** 1 University Hospital of the RWTH Aachen University, Section Neuropsychology – Department of Neurology and Interdisciplinary Centre for Clinical Research (IZKF "BIOMAT."), Germany; 2 Department of Psychology, Paris-Lodron University Salzburg, Austria; 3 Department of Psychology, Eberhard Karl University Tübingen, Germany

## Abstract

FMRI data observed under a given experimental condition may be decomposed into two parts: the average effect and the deviation of single replications from this average effect. The average effect is represented by the mean activation over a specific condition. The deviation from this average effect may be decomposed into two components as well: systematic variation due to known empirical factors and pure measurement error. In most fMRI designs deviations from mean activation may be treated as measurement error. Nevertheless, often deviation from the average also may contain systematic variation that can be distinguished from simple measurement error. In these cases, the average fMRI signal may provide only a coarse picture of real brain activation. The larger the variation within-condition, the coarser the average effect and the more relevant is the impact of deviations from it. Systematic deviation from the mean activation may be examined by defining a set of parametric regressors. Here, the applicability of parametric methods to refine the evaluation of fMRI studies is discussed with special emphasis on (i) examination of the impact of continuous predictors on the fMRI signal, (ii) control for variation within each experimental condition and (iii) isolation of specific contributions by different features of a single complex stimulus, especially in the case of a sampled stimulus. The usefulness and applicability of this method are discussed and an example with real data is presented.

## Background

We present an update about the use of a special type of parametric designs in fMRI research that can be very useful in investigations involving natural and multi-featured stimuli such as pictures or words. This method has already been developed by Büchel and colleagues [1] but unfortunately it has not been used as frequently as it deserves. For this reason, we present a summary of the logic behind the use of parametric designs in fMRI research, discuss shortly its mathematical background and applicability, and present an empirical example where parametric regressors carry the most relevant modulation of the fMRI signal.

In several fields of neurocognition, stimuli can be assigned to experimental conditions so that they (i) are homogeneous within each cell of an experimental design and (ii) differ only with regard to a single aspect across the different levels of an experimental factor. Each statistical contrast unequivocally isolates therefore one and only one neurocognitive process. However, in the case of natural stimuli, such as pictures (e.g. kitchen utensils vs. garage tools) or written words (e.g. varying in length, number of syllables, frequency, neighbourhoods, regularities, consistencies etc.), the task of matching different groups of items for their attributes is particularly challenging, because there is often only a finite a number of stimuli to fit into each of the different cells of the experimental design that vary simultaneously in *more than one *feature. In these cases, different dimensions of stimuli can only be matched on average. Words, for instance, can vary in the number of letters, the frequency of occurrence, the number of lexical neighbours as well as the frequency of occurrence of orthographic or phonological sub-lexical units. Different words may have for example 1, 2 or 8 different lexical neighbours. Therefore, for each stimulus dimension (i.e. frequency of occurrence, number of lexical neighbours, etc.) there is a non-zero distance between each single item and the average for each of the different dimensions, characterizing the amount of variation within condition.

Due to variation within condition, the statistics for the size of fMRI signal elicited by the different items pertaining to the very same condition may vary considerably. Consequently, the type-II error for detecting a difference in fMRI signal between two different conditions may be inflated. The main problem for the interpretation of the results of such an experiment is whether it is acceptable to consider the variation within condition as measurement error or not. If the variation within condition is small in comparison with the variation between different conditions, treating it as measurement error is not problematic. However, if the variation within a cell of the experimental design increases due to systematic variation in known dimensions of multi-featured stimuli, the validity of the whole study may be questioned.

In the present paper we examine a method proposed by Büchel and colleagues [1] for dealing with systematic variation between items. The method involves the definition of parametric regressors representing each of the several dimensions of complex stimuli. These parametric regressors absorb the systematic variation inherent in different dimensions of complex stimuli such as words, sentences or arithmetic problems, and allow for separating it from genuine measurement error. In the following, we will present the method, discuss its main applications, and present an example in which the variation between items (and their exact scaling properties) was the most relevant aspect of the experimental design.

### Overview of the method

Activation *Y*_*ij *_in a particular voxel can be described as in (1) for each replication *i *(for every *i *from 1 to *p*) of an experimental condition *j *(for every *j *from 1 to *q*):

(1) Y_ij _= *α *+ *β*_j_X_j _+ *ε*_ij_

having *a *as the intercept, *X*_*j *_as a (continuous) parameter describing the present experimental design, *β*_*j *_as the regression coefficient for the parameter *X*_*j *_and *ε*_*ij *_as residual error. The coefficient *β*_*j *_describes the event- or block-specific expected BOLD-response under a given experimental condition *j *assuming that within an experimental condition the BOLD-response induced by event- or block-specific stimulation will be a constant. A corollary of this assumption is that variation in the BOLD response occurring within an experimental condition will be considered residual error.

When stimuli in an experimental condition are sampled from a universe of natural items, some variation among items will always be present. An artificial increase of residual error *ε *due to variation in the BOLD-response produced by variation within condition contributes negatively to the sensitivity of the fMRI design. Importantly, when the variance among items not only represents a confounding factor but genuine scaling properties of stimulus features, it is mandatory to deal with them appropriately by modelling this variance within conditions.

Parametric modelling always allows for the description of variation in the event- or block specific BOLD-response, when the source (or sources) of variation is known *a priori *and can be specified numerically as parametric regressor. Importantly, the variation within conditions may be due not only to one single stimulus feature, but rather be due to two or more features. In this case, for each of the dimensions of multi-featured stimuli a regressor can be defined, which absorbs the contribution of that dimension for the variation within a given experimental condition (but see the section on the limitations of this approach in the discussion, below). The specification of parametric regressors is given as follows: the parameter *X*_*j *_described by a canonical hemodynamic function in common fMRI designs, which has exactly the same form across all replications *i *of a given experimental condition *j*, can be expressed as the average effect *X*_*j *_of a predictor *X *on brain activation. Moreover, in parametric designs a second set of predictors may be complemented by a set of *k *dimensions (for every *k *from 1 to *r*) which are *nested *under each condition j and which absorb the variation within each condition. The full model presented in (2) contains a predictor representing the average effect of experimental condition *j *plus an additional parameter for each parametric regressor *k *considered. *X*_*ij*1_, *X*_*ij*2_, ... *X*_*ijk *_... *X*_*ijr *_contain the variation in each of k different stimulus dimensions. Note that parameters *β*_*jk *_are hierarchically bound to the average parameter *β*_*j *_and that the number of parameters *β*_*jk *_associated with each average parameter *β*_*j *_may differ. Therefore, (1) can be generalized by assuming a set of *r *> 1 dimensions:

(2) Y_ij _= *α *+ *β*_j_X_j _+ *β*_j1_X_ij1 _+ ... + *β*_jk _X_ijk _+ ... +*β*_jr_X_ijr _+ *ε*_ij_

By entering parametric regressors in the fMRI design, the proportion of variance which can be accounted for by the variation within conditions is separated from the residual error *ε*_*ij*_. This extension of the model presented in (1) has two consequences: (i) the statistical test on the significance of null-order parameter *β*_*j *_will not be biased by variation within conditions, which can be explained by predictors *β*_*j*1 _to *β*_*jr*_. (ii) Furthermore, the relevance of regression coefficients *β*_*j*1 _to *β*_*jr *_may be assessed.

The definition of parametric regressors with the single purpose of isolating variation within conditions as a confounding factor is trivial and has been employed regularly in fMRI research. The sole purpose of this application is to control for the impact of undesired sources of variance affecting statistics about the effects of interest. In this case, variation within conditions can be considered an effect of non-interest, the impact of which on the statistics can be partialled out from residual error.

Nevertheless, parametric regressors also may be defined with the aim of directly investigating theoretical predictions with respect to the fMRI activation observed. In the following, we will concentrate on the advantages and limitations of such an application. In fact, parametric regressors make possible an investigation of the direction and actual scaling properties of variation of fMRI activation. Examination of the impact of quantitative regressors on the fMRI activation has been presented by Büchel and colleagues [1]. In that study the authors defined one single parametric regressor and applied polynomial expansions (i.e. quadratic, cubic, etc.) to investigate non-linear relationships between the BOLD-response and this single experimental parameter. Here we use the method [1] for two purposes: (i) instead of examining the impact of polynomial expansion of a single parametric predictor on fMRI activation, defining a set of predictors which, according to some theoretical expectation, may account for a significant amount of variability among trials produced by known and quantifiable properties of stimuli. Furthermore, the method is useful for (ii) assessing the significance of each single parameter for brain activation (i.e. one-sample t-tests) to the comparison between different models (i.e. statistics for two or more samples), which normally differ only with respect to one out of a set of parametric regressors. With this second type of application, we are able to statistically test hypotheses about the exact form of variation in fMRI activation.

In the following example, we compare the model fit obtained for different numerical compressions of the predictors employed (i.e. logarithmic vs. linear scale). Results of these comparisons may help to determine the exact form of variation and the underlying rate of neuronal response to each of the different stimulus dimensions examined.

### An empirical example

Numerical cognition provides a straightforward example for the usefulness of parametric regression. Numbers do naturally differ in their parametric properties, such as, for instance, their magnitude [2-4]. Since no number shares the same magnitude with another, naturally there is variation in this dimension within every experimental condition in which different numbers are used. Number magnitude is assumed to be represented in the cortex around the intraparietal sulcus (IPS) [2,3]. Behavioral studies [5] and a neural network model [6] have indicated that numerical distance is logarithmically compressed. Some recent single-cell recording studies reported that cells in pre-frontal and parietal cortex are tuned to specific magnitudes [7-11]; their signal is best described by a logarithmically compressed scale [10,11]. Similar results have been obtained in fMRI studies [12,13]. Furthermore, studies on two-digit number processing have shown that participants may not be able to compare the magnitude of decade digits while ignoring the unit digits, even when the units are totally irrelevant for the task at hand [see [5,14,15] for a review, [16,17]].

Given this theoretical background, we ask two empirical questions about the fMRI signal that can be investigated more precisely by means of parametric than by conventional categorical methods. The first question is whether the fMRI signal in the intraparietal cortex is better accounted for by the overall distance when participants are asked to choose the larger from two two-digit Arabic numbers or by the distance between decade digits. Since there are no two-digit numbers "without" a unit digit to serve as stimuli for a control condition, the only way to examine this problem empirically is to compare the BOLD-response evoked by overall distance with that evoked by decade distance (decade digit_larger number _– decade digit_smaller number_). If the statistical fit for overall distance is better than for decade distance, one may infer that the fMRI activation in the intraparietal cortex due to the contrast (overall distance > decade distance) is associated with the processing of the overall magnitude of numbers.

A second empirical question is whether the fMRI signal in intraparietal cortex is better accounted for by the logarithm of the distance than by the linear distance between two-digit numbers. This question has been investigated first in an fMRI study by Pinel and colleagues [4]. These authors found that in six out of seven regions of interest the percent signal change dropped in accord with the logarithm of the distance between numbers rather than with the linear distance. Nevertheless, the authors examined the effect of logarithmic scaling on fMRI signal by splitting the range of distances into three arbitrary categories (i.e. small, medium and large distances) instead of treating distance as a continuum. This approach presents disadvantages in comparison with the modelling with parametric regressors: The method employed by Pinel and colleagues [4] may fail to distinguish between the impact of decade distance and overall distance on fMRI signal (i.e. the first empirical question examined in the present example). This may have affected the determination of the exact spatial distribution of the neurons responding more strongly to the logarithmically compressed magnitude of numbers.

In the following, we describe the results of the parametric analysis of an fMRI experiment examining the two empirical questions stated above.

#### Procedure

Fourteen male right-handed volunteers (mean age = 27, range 21–38 years) took part in the study after giving their written consent to the imaging protocol which has been approved by the local Ethics Committee of the Medical Faculty and is in compliance with the Helsinki Declaration. Participants had to select the larger number of a pair of two-digit Arabic numbers (range: 21–98) and press a key [for further details on the design of experiment and characteristics of the task as well as on behavioural data, see [16], including supplementary material]. Overall distance, decade distance, unit distance and problem size were matched both absolutely and logarithmically between stimulus categories [16]. The four digits chosen as units and decades of the two-digit number pair were always different. Furthermore, in the present study unit numbers were totally irrelevant for magnitude comparison since no within decade comparisons were included.

#### MRI acquisition

For each participant, a high-resolution T1-weighted anatomical scan was acquired with a Philips 1.5T Gyroscan MRI system (TR = 30 ms, matrix = 256 × 256 mm, 170 slices, voxel size = 0.86 × 0.86 × 2 mm; FOV = 220 mm, TE = 4.6 ms; flip angle = 30°). The anatomical scans were normalized using the standard T1 template of SPM2.

#### fMRI acquisition

Two functional imaging runs sensitive to blood oxygenation level-dependent (BOLD) contrast were recorded for each participant with a Philips 1.5T Gyroscan MRI system (T2*-weighted echo-planar sequence, TR = 2800 ms; TE = 50 ms; flip angle = 90°; FOV = 220 mm, 64 × 64 matrix; 30 slices, voxel size = 3.4 × 3.4 × 4 mm). In each run, 316 scans + 5 dummy scans were acquired. In a rapid event-related design, a total of 576 trials (480 experimental trials + 96 null events) were presented at a rate of 3 seconds. The fMRI time series was corrected for movement and unwarped in SPM2. Images were resampled every 4-mm and normalized to a standard EPI template using the sinc interpolation method. Moreover, functional images were co-registered with the normalized anatomical pictures. Finally, functional images were smoothed with an 8-mm Gaussian kernel.

#### Parametric design

We convolved brain activity for all experimental trials with the canonical hemodynamic response function (HRF) in *a single experimental condition *and defined three parametric regressors representing overall distance, decade distance, and problem size. The correlations between the different parameters and the in-line correlations (i.e. the correlations obtained after convolution with the HRF function) between the parametric regressors and the average hemodynamic response are shown in Table 1 and Table 2, respectively. In order to scale the estimated regression parameters uniformly, the parametric regressors representing overall distance, decade distance, and problem size were standardized to a mean of 0 and a standard deviation of 1.

**Table 1:** Means and correlation matrix for the parametric regressors (n = 240 items, variances in the main diagonal)

	dist10	logdist10	dist	logdist	size
dist10	**391**				
logdist10	0.97	**0.09**			
dist	0.98	0.95	**377**		
logdist	0.95	0.96	0.97	**0.08**	
size	-0.03	-0.05	-0.03	-0.05	**652**

mean	36.63	1.48	36.72	1.49	118.43

dist10: decade distance, logdist10: base-10 logarithm of decade distance, dist: overall distance, logdist: base-10 logarithm of overall distance, size: problem size

**Table 2:** In line correlation matrix for the parametric regressors

	Model 1 "overall distance"		Model 2 "decade distance"	
	Overall distance	Problem size	Decade distance	Problem size

Average BOLD function	-0.05		-0.05	
Overall distance	-0.03	-0.03	-0.03	-0.02

In order to examine whether the fMRI signal in the intraparietal cortex can be better accounted for by the overall distance than by decade distance alone, we estimated two separate models. In one model, overall distance and problem size were entered as parametric regressors and, in a separate model, decade distance and problem size were entered as parametric regressors. A summary of the procedure for definition, estimation, and statistical assessment of the different parametric models is presented in Table 3.

**Table 3:** Summary of model definition, estimation and statistical comparison using parametric predictors

		Model 1	Model 2
	Model name	"overall distance"	"decade distance"

First level (coefficient estimation)	predictors	"overall distance" , "problem size"	"decade distance" , "problem size"
Second level Paired two-sample t-tests		"overall distance" > "decade distance"	
		"overall distance" < "decade distance"	

	Model name	"log-overall distance"	"overall distance"

First level (coefficient estimation)	predictors	"log overall distance", "problem size"	"overall distance", "problem size"
Second level Paired two-sample t-tests		"log-overall distance" > "overall distance"	
		"log-overall distance" < "overall distance"	

### ROI analysis

To avoid the problem of multiple comparisons typical for whole brain analysis when assessing the empirical hypotheses about the amount of signal captured by parametric predictors, small volume analysis was carried out in specific sub-regions of parietal cortex. For the analysis of the regions of interest (ROI), 6 mm-spheres in the left and right parietal cortex were extracted from the brain images using the toolbox MARSBAR. Selection of these ROIs was based on regions showing significant differences in the experimental contrasts in the whole brain analysis.

## Results

**Figure 1:**
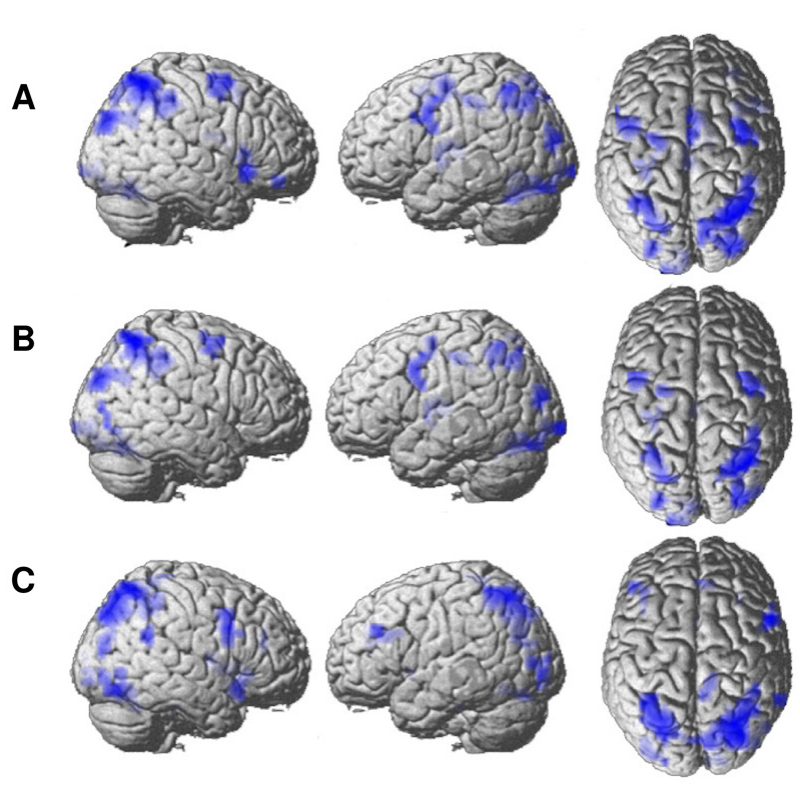
Activation produced by (A) logarithm of overall distance, (B) overall distance and (C) problem size (*p *< .001, uncorrected, k = 10 voxels).

**Figure 2:**
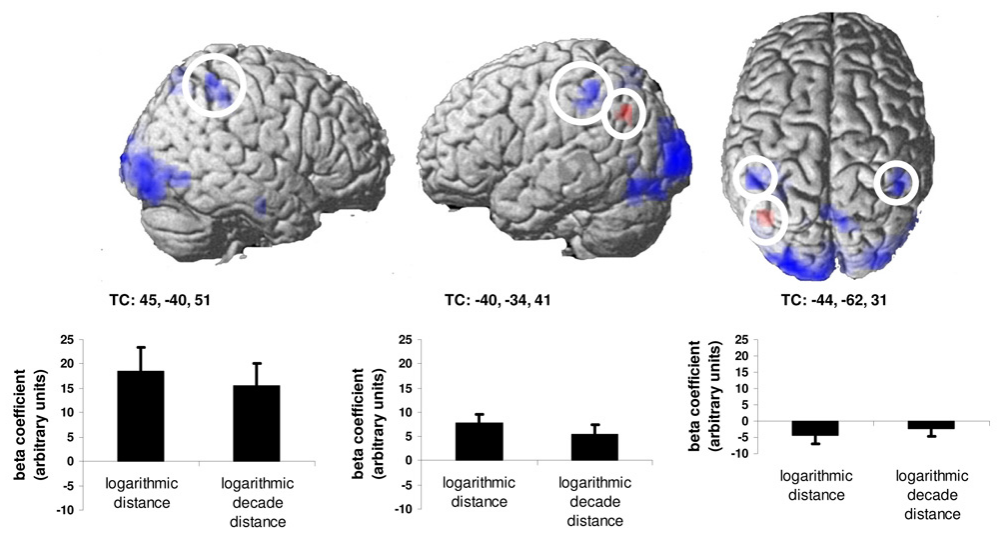
**Voxels showing stronger activation for the overall distance than for decade distance are coloured blue while voxels showing stronger activation for the decade distance than for overall distance are coloured red (*p *< .001, uncorrected, k = 10 voxels).** While overall distance activated large portions of the anterior intraparietal cortex bilaterally in comparison with decade distance only, as well as in the extrastriate cortex, decade distance only deactivated voxels in the left angular gyrus relative to overall distance. ROI analyses revealed stronger activation in the intraparietal cortex bilaterally in response to overall distance compared to decade distance as well as a slight deactivation in the left angular gyrus.

One-sample t-contrasts revealed that overall- and decade distance as well as problem size predicted activation in parietal cortex – especially in the cortical regions in the vicinity of the intraparietal sulcus – as well as in occipital, premotor, and prefrontal cortices (Figure 1). Interestingly, the paired two-sample contrast "overall distance > decade distance" revealed strong fMRI activation in the intraparietal cortex (Table 4). ROI analyses pointed out that significantly more activation in response to overall distance than for decade distance was found in the left and right parietal cortex centered at Talairach coordinates x = -40, y = -34, z = 41 (*t*(13) = 5.54; *p *< .001) and x = 45, y = -40, z = 51 (*t*(13) = 4.21; *p *= .001; Figure 2). The contrast "decade distance > overall distance" revealed no activation in intraparietal cortex but only a slight deactivation in the left angular gyrus. ROI analysis revealed a significant deactivation for logarithmic decade distance in comparison with logarithmic overall distance in the left angular gyrus (x = -44, y = -62, z = 31; *t*(13) = -4.63; *p *< .001; Figure 2).

**Table 4:** Brain areas activated more by overall distance or decade distance, respectively

Overall distance > decade distance				
Region	Talairach coordinates x, y, z§	*t*-value df = 13	BA	Cluster size k

Left extrastriate cortex	-24, -93, 8	10.10**	19	356
Right extrastriate cortex	32, -86, -2	10.02**	19	-
Left anterior intraparietal cortex	-51, -33, 38	8.39**	40	32
Left striate cortex	-16, -66, 7	5.87**	40	15
Right anterior intraparietal cortex	44, -37, 42	5.15**	40	20
Left superior parietal lobule	-4, -60, 51	6.07**	7	18
Right fusiform gyrus	36, -13, -20	5.17**	19	10

Decade distance > overall distance				

Left angular gyrus	-44, -60, 33	-5.38*	39	10

Logarithmic overall distance > overall distance				

Left posterior intraparietal cortex	-33, -52, 44	7.42**	7	75
Left anterior intraparietal cortex	-32, -30, 41	5.61**		40
Left extrastriate cortex	-28, -84, 18	6.18**		26
Left premotor cortex	-24, -5, 48	7.49**		26
Left premotor cortex	-43, 1, 44	7.07**		46
Right posterior intraparietal cortex	37, -56, 50	8.45**	7	234
Right anterior intraparietal cortex	40, -33, 41	7.65**	-**a**	40
Right frontal operculum	34, 18, 10	7.21**		21
Right SMA	4, 7, 51	6.41**		29
Right ventrolateral prefrontal cortex	45, 16, -2	6.06**		21
Right premotor cortex	44, -1, 49	6.05**		34
Right orbitofrontal cortex	31, 39, -9	5.80**		16
Overall distance > logarithmic overall distance				
No suprathreshold clusters				

§ transformed from the MNI coordinates with the SPM tool mni2tal; ** p-value at the cluster level < .05, corrected; **a**: local maximum in the same cluster as the right posterior intraparietal cortex

To examine whether the fMRI signal in the intraparietal cortex could be better accounted for by the logarithm of the distance than by the linear overall distance between the two two-digit numbers, we estimated two separate models: In one model, overall distance was entered as a parametric regressor; in a separate model, the logarithm of overall distance was employed as a parametric regressor. Both linear and logarithmic overall distance were significant predictors of activation in intraparietal cortex. Interestingly, the logarithmic overall distance was a better predictor of fMRI activation in a broad network of brain regions including the right and left posterior intraparietal cortex, left anterior intraparietal cortex, left extrastriate cortex, left premotor cortex, right frontal operculum, right SMA, right ventrolateral prefrontal cortex, right premotor cortex and the right orbitofrontal cortex (Table 4). In the contrast "linear overall distance > logarithmic overall distance" no activation was observed at the threshold of *p *= .001, uncorrected, k = 10.

To examine the differential impact of logarithmic overall distance on the MRI activation in IPS, four ROIs were extracted for the contrast "logarithmic overall distance > linear overall distance". Posterior left (x = -32, y = -53, z = 44, *t*(13) = 7.15; *p *< .001) and right intraparietal cortex (x = 37, y = -56, z = 49; *t*(13) = 8.10; *p *< .001) as well as left (x = -52, y = -46, z = 38; *t*(13) = 5.97; *p *< .001) and right anterior intraparietal cortex (x = 40, y = -33, z = 41; *t*(13) = 7.10; *p *< .001) were activated more strongly by logarithmic than by linear overall distance.

## Discussion

**Figure 3:**
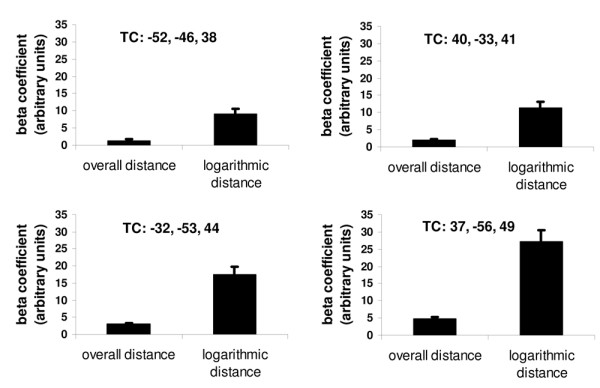
**ROI analysis of the contrast logarithmic overall distance > linear overall distance.** Both, in the anterior and posterior intraparietal cortex, bilaterally, more activation was found for the logarithm of overall distance.

In the present paper we have examined the applicability of the parametric methods presented in [1] in two ways: (i) the specific impact of each one out of a set of parametric regressors on fMRI activation (Figure 1) and (ii) the comparison of different quantitative models of the scaling properties of fMRI activation (Figures 2 and 3). Using parametric modelling of fMRI data, we have shown that the hemodynamic response in the intraparietal cortex, bilaterally, is sensitive to the overall magnitude of two-digit numbers (decade + unit distances), since the parametric model containing overall distance predicted fMRI activation significantly better than the model containing decade distance only. These results indicate that participants are not able to ignore the magnitude of unit digits when comparing two-digit numbers: unit magnitudes are processed – behaviorally as well as neurofunctionally – even if they are irrelevant for the comparison [4,5,14-17]. Furthermore, by examining the influence of decades and units on brain activation, we found that the left angular gyrus was deactivated more in response to decade distances than to overall distances. Commonly, deactivation of the angular gyrus is interpreted as the product of an enhancement of visuospatial attention [18]. In the present case we tentatively interpret the stronger deactivation in response to decade distances as a product of the effort implied in the selection of just the decade distances for comparison. Since the correct result for the magnitude comparison could be reached in the experimental task by comparing the decade digits alone, participants may have tried to process their magnitude in more detail than unit magnitudes. For doing so, they need more visuospatial attention to select decade digits in the visual display. To our knowledge this is the first report of that effect, which should be investigated in further studies.

Together, results also support the view that number magnitude is represented in the intraparietal cortex in a logarithmically compressed fashion, that irrelevant unit magnitudes determine fMRI activation, and that participants may engage visuospatial attention in order to select decade digits for processing. In general, the present data are in line with an extensive behavioural and fMRI literature [[2,4,5,12,14], and [3] for a review].

In the following, we will discuss the relevance of the present results as an illustration of the advantages of modelling the fMRI signal with parametric regressors. Specifically, three points will be emphazised: (i) the impact of continuous predictors on fMRI signal, (ii) the control of variation within experimental conditions due to known features of complex stimuli and (iii) the isolation of specific contributions by quantifiable features of single complex stimuli, especially in the case of stimuli sampled from a pool of natural items. In the final section we will discuss some limitations of the parametric method.

### Continuous predictors of fMRI signal

The most important feature of the parametric method is the modelling of variation in different dimensions of natural stimuli in a natural way that is not constrained by the necessity of generating (sometimes arbitrary) categories of stimuli in order to look at average differences between these categories. Therefore, no categorization of distances is necessary when comparing for instance linear and logarithmic scales, since the distances themselves are entered in the model as predictors of activation.

One could argue that instead of employing parametric modelling, the experimenter could mask the decade or unit digit of different numbers in order to isolate the effects of overall magnitude and decades in the different experimental conditions. This approach is, however, problematic, since it is not clear whether the single digits at the decade and unit positions in two-digit numbers also have their own magnitude representation. For this reason, presenting digits of a two-digit number separately from each other would possibly lead to activation of separate magnitudes [17,19] and cannot be considered as a valid test about two-digit number processing. With parametric regressors, the selective contribution of one stimulus feature can be assessed in experimental settings in which other stimulus features cannot be held constant without destroying their usual perceptual or semantic structure. Among these features, which cannot be held constant, one may differentiate between those which are of experimental interest and those which are simply confounds without any special theoretical meaning. This latter aspect of sampled stimuli will be examined in the next section.

### Variation of interest and of no-interest within an experimental condition

As mentioned in the overview about this method, parametric regressors may represent the only source of variance of interest in an experiment. In the empirical example, parametric regressors were shown to be suited for testing non-trivial empirical hypotheses about fMRI signal. Parametric regressors were not entered in the model only in order to control for known sources of inhomogeneity, but they were the actual experimental factor.

As already pointed out in [1], the experimenter may isolate linear and non-linear contributions of the same predictor to the BOLD-response. In this sense, parametric models may be able to better approximate scaling properties of activity in very specialized groups of neurons. The empirical hypotheses tested in the present study involved predominantly the scaling of the fMRI signal (linear vs. logarithmic distances). Comparing the relative fit obtained by modelling data with linear predictors vs. logarithmically compressed regressors was made possible by employing parametric models. The aim of modelling data with linear vs. logarithmic predictors was to compare the relative fit obtained by modelling fMRI signal either with linear overall distance or with the logarithm of overall distance. As illustrated by the present results, the parametric method allows for detecting even very subtle details of the scaling properties of the BOLD response. Inspection of Table 1 reveals that the parametric regressors representing overall distance and decade distance are highly correlated (*r*(240) = .96, *p *< .05). This means that the advantage of overall distance in predicting fMRI activation in the intraparietal cortex bilaterally is due to fine-grained differences in the scaling properties of regressors representing overall distance and decade distance, which can only be captured in a parametric model. Voxels significantly better tuned to the logarithm of overall distance than to the overall distance itself could be observed in the intraparietal cortex. Similar arguments can be put forward for comparing the relative response of voxels in the angular gyrus to decade distance and overall distance. Our analyses suggest that overall distance rather than decade distance is generally more closely related to IPS activation while decade distance led to deactivation of the left angular gyrus.

As mentioned before, in parametric analyses, predictors may represent different features of stimuli, which, even being theoretically different constructs, may selectively contribute to the activation in a single voxel. Importantly, the covariance structure between different predictors should be taken into consideration when designing a parametric fMRI study. In the optimal case the parametric predictors should be orthogonal to each other. If this condition cannot be reached in a specific case, the correlations between the different predictors should be held low (see the section on the limitations of parametric models, below). Furthermore, the number of predictors entering a parametric model should be much smaller than the number of scans in the individual time series from which the beta coefficients for each condition are estimated. In general this is not problematic, since the number of scans acquired for reach participant is very large in comparison with the number of experimental conditions of interested.

In the example presented above, the activation produced by numerical distance and by problem size in a given voxel could be disentangled using the parametric model: Both overall distance and problem size activated highly overlapping regions in the intraparietal cortex bilaterally. Nevertheless, there is no doubt that overall distance and problem size remain different features of two-digit numbers. We also have demonstrated a selective increase in fMRI signal in the intraparietal cortex which was better explained by the overall distance than by decade distance alone. Moreover, we also found a small cluster of voxels in the left angular gyrus, which responded more strongly to decade distance alone rather than to overall distance (Figure 2). Only with parametric regressors it was possible to "extract" and assess the selective contribution of the decades for the fMRI signal to a complex stimulus, which by commonly activates different perceptual, motor and cognitive representations [3].

### Isolating the contribution made by different features of a single complex stimulus

Natural stimuli are complex objects which carry different perceptual as well as different conceptual features. These features may present variation across the different objects selected to form a category of objects in an empirical investigation. Ollinger and colleagues [20] presented a method for separating processes within a trial in event-related fMRI designs. The authors have shown that perceptual, cognitive, and motor processes may be confounded in complex tasks. They presented a method for isolating the relative impact of each one of these single processes by means of a manipulation of the sequence of trials. One important precondition for employing this method is that the different processes activated within a trial can be assessed separately. Unfortunately, this assumption is not valid for all experimental designs. In the domain of number processing, examination of the relative impact of decade and unit digits on brain activation cannot be investigated in a natural way without presenting decade and unit digits in every trial, for there is no two-digit number without a decade or a unit digit. In parametric models the impact of different features of a complex stimulus can be modelled simultaneously, their specific effects can be isolated from the effects of other features, and most importantly, the specific effect of each feature on the activation observed in a given voxel can be assessed statistically. In the example presented above, the amount of signal produced by problem size was controlled for in the different analyses, since problem size was entered as a second predictor in every model examined. In the present case, the correlation between regressors representing those numerical distances and that representing problem size was not different from 0 (Table 1). Therefore, the effect of problem size on fMRI activation did not interfere substantially with the impact of overall and decade distances on fMRI activation.

However, in specific applications the correlation between different stimulus dimensions may differ from 0. In such cases it is imperative to define the different stimulus dimensions in the same model, in order to isolate the specific contribution of each dimension to the fMRI activation and to obtain the correct statistics regarding each of these correlated dimensions.

Parametric designs are not a substitute for the careful selection of items for an empirical investigation. The correlation matrix of the different properties of stimuli is the most adequate index for assessing the suitability of parametric modelling of fMRI data. When the correlations between different predictors are moderate or high, their interpretability as separate conceptual entities is compromised. For this reason, the selection of adequate predictors for a parametric study may turn out to be a non-trivial task, since the correlations between the different parametric properties of items, which are controlled for in a given empirical investigation, should be held small or even non-significant. In the present study, we were able to isolate the impact of overall distance and problem size on fMRI signal because the correlation between these two properties of items could be kept very low (*r*(240) = -.03, n.s.). Accordingly, in the study by Wood and colleagues [16] the selective impact of decade and unit distance could be well separated in a parametric analysis since the correlation between the parametric regressors representing them was low, too (*r*(240) = .18, *p *< .05).

The only case in which parametric modelling with highly correlated predictors is informative is the comparison between the relative fit of *two *different models, one containing one of the two predictors and the other model containing the other one. A paired two-sample t-test may reveal whether one of these predictors explains more variance of fMRI signal (see the section on linear vs. logarithmic scaling of overall distance, above). In this case, the only difference between the two models should be produced by the scaling of the parametric predictor. Neurons in intraparietal cortex bilaterally respond to the magnitude of two-digit numbers in a logarithmically compressed fashion. This piece of evidence is in line with current theories of number magnitude processing [3] and with evidence from behavioural [14], fMRI [4,12] as well as single-cell recording studies [7-11]. Moreover, a discussion on the compression of magnitude representation has been put forward by Dehaene [6], who argues that the magnitude representation may assume a more linear scaling with training in arithmetical tasks. The question whether the neural response in the intraparietal cortex also changes from a logarithmically compressed scaling to a more linear one can be directly assessed with parametric models, indicating that the parametric method represents not only an alternative method for data analysis but also a tool for testing specific empirical hypotheses with more precision.

### Some limitations

#### Multi-collinearity

As in every implementation of the general linear model, only the orthogonal part of the variance of a parametric regressor has its impact on fMRI signal tested for statistical significance [21]. When the different parametric regressors are highly correlated, the orthogonal part of each parametric regressor may become very small and lose empirical relevance. For this reason, the interpretation of parametric designs in the presence of multi-collinearity is problematic. Before carrying out a parametric fMRI study, a careful selection of items should be conducted in order to avoid large correlations between the predictors of interest. In any case, the correlations between the different predictors in an fMRI design should be inspected and reported in the manuscript.

#### "Deactivation" and inverted contrasts

Since the parametric regressors represent the deviation of single items from the average of the experimental condition and not the average activation itself, the notion of "deactivation" must be viewed differently in parametric models. "Deactivation" in a paramentric regressor means that the direction of the association between variation within condition and fMRI signal is inverted.

## Conclusion

The parametric method can be very useful for investigations involving complex stimuli characterised by several different features. The more complex the tasks (e.g. complex arithmetic tasks or reading words from very specific word classes), the more adequate is the parametric modelling of stimulus features, since in many occasions authentic variation in stimulus properties cannot be matched exactly between different conditions, but only on average. In these cases genuine variance within conditions will be present and should be treated as such and not as measurement error. Modelling fMRI data using parametric regressors allows for the simultaneous quantification of variation in many stimulus dimensions and may be very useful for simultaneously isolating and statistically assessing the contribution of variation in different dimensions. However, the careful choice of items in each experimental condition cannot be substituted by adding parametric regressors to the statistical models at the high cost of interpretability of results. Moreover, the correlations between the different parametric predictors entering the statistical model should be zero or close to zero. The only exception from this rule is the comparison between linear and non-linear transformation of the same parametric predictor. Finally, when the (statistical) assumptions for their use are fulfilled, parametric models represent a very useful tool for assessing empirical hypotheses in fMRI studies more precisely.

## Competing interests

The authors declare that they have no competing interests.

## Authors' contributions

GW Participated in the design of the study and carried out behavioral and fMRI measures and statistical analyses, DS Carried out the region-of-interest analyses, HCN and KW participated in the conceptual formulation of the research question and the design of the study. All authors read and approved the final version of the manuscript.
